# Relevance of erythrocyte sedimentation rate and C-reactive protein in patients with active uveitis

**DOI:** 10.1007/s00417-018-4174-7

**Published:** 2018-11-08

**Authors:** Fahriye Groen-Hakan, Laura Eurelings, Jan van Laar, Aniki Rothova

**Affiliations:** 1 Department of Ophthalmology, Erasmus Medical Center, Erasmus University, Rotterdam, the Netherlands; 2 Department of Internal Medicine and Department of Immunology, Erasmus Medical Center, Erasmus University, Rotterdam, the Netherlands

**Keywords:** Erythrocyte sedimentation rate, C-reactive protein, Uveitis, Diagnostics

## Abstract

**Purpose:**

To relate erythrocyte sedimentation rates (ESR) and C-reactive protein (CRP) values to different uveitis entisties.

**Methods:**

A retrospective study of patients with a first episode of active uveitis visiting the Erasmus University Medical Center, uveitis clinic, Rotterdam, the Netherlands, was performed. Levels of ESR and CRP were determined within 2 weeks and 1 week after onset of uveitis, respectively. Uveitis had to be of unknown origin at that moment. The specific etiologic groups were related to ESR and CRP values.

**Results:**

The majority of patients with uveitis had ESR and/or CRP values within the normal limits and no association of ESR and/or CRP with the specific cause of uveitis was observed. However, elevation of ESR ≥ 60 mm/h and/or CRP ≥ 60 mg/L was mostly seen in patients with systemic immune-mediated diseases (8/59, 14% of all with immune-mediated diseases) or systemic infectious causes (7/38, 18% of all infectious uveitis). Patients with ocular toxoplasmosis typically exhibited normal ESR and CRP (9/11, 82%) while patients with endogenous endophthalmitis had elevated ESR and/or CRP in 6/7, 86%. Sarcoidosis-associated uveitis showed predominantly elevated ESR (13/24, 54%; range 20–59 mm/h in 11/13, 85%). Human immunodeficiency virus–positive patients had more often elevated ESR values when compared to the remainder of patients (9/11, 82% vs. 64/163, 39%, 18%, *P* = 0.009). The cause of uveitis was established in 19/20 (95%) of patients with ESR ≥ 60 mm/h and/or CRP ≥ 60 mg/L.

**Conclusions:**

The majority of patients with first attack of uveitis had ESR and CRP within the normal limits. Elevated levels of ESR and CRP reflected systemic involvement and high levels of both values were associated with established uveitis cause.

**Electronic supplementary material:**

The online version of this article (10.1007/s00417-018-4174-7) contains supplementary material, which is available to authorized users.

## Introduction

Uveitis is an intraocular inflammation of multiple causes, which may result in permanent visual loss [1–4]. Erythrocyte sedimentation rate (ESR) and C-reactive protein (CRP), both non-specific markers of inflammation, are usually included in the initial diagnostic workup. However, the clinical value of these parameters in the adult uveitis population is not known. Earlier investigations showed that ESR and CRP are within the normal range in a majority of patients with anterior uveitis [5]. In contrast, a recent report on juvenile idiopathic arthritis (JIA)-associated uveitis in a pediatric population showed that elevated ESR predicted the development of uveitis in patients with JIA [6]. However, it remains debatable whether ESR and CRP have any diagnostic value in evaluation of uveitis in adult patients having a first uveitis attack of unexplained origin.

Herein, we investigate the values of ESR and CRP during the first episode of active uveitis, determined within a short period after the onset in adult patients and relate the results to specific etiologic categories and clinical characteristics of uveitis.

## Materials and methods

The Strengthening the Reporting of Observational studies in Epidemiology (STROBE) guidelines were used to ensure the reporting of this observational study and this study followed the Tenets of the Declaration of Helsinki [7].

We conducted a retrospective cross-sectional study at the ophthalmology department of the Erasmus Medical Center (Rotterdam, the Netherlands). All medical records of patients referred with new uveitis of unknown origin investigated between 2010 and 2017 were reviewed and 174 patients were identified who fulfilled our inclusion criteria. The onset of uveitis was defined as the first time active inflammation was documented by an ophthalmologist. ESR had to be determined ≤ 2 weeks and CRP values ≤ 1 week after the onset uveitis (as ESR normalizes within weeks and CRP levels within 7 days after resolution of tissue injury) [8]. Exclusion criteria included age less than 18 years and patients with first mild anterior uveitis episode as these patients do not undergo diagnostic screening according to our guidelines. The ESR and CRP values were arbitrarily stratified to three subgroups (normal ESR < 20 mm/h, elevated ESR between 20 and 60 mm/h, highly elevated ESR ≥ 60 mm/h and normal CRP < 10 mg/L (as defined in our laboratory), elevated CRP between 10 and 60 mg/L and highly elevated CRP ≥ 60 mg/L).

The following data were extracted from patients’ records: age, gender, localization of uveitis, laterality, and human immunodeficiency virus (HIV) status. All immunosuppressive medications as well as co-morbidities were registered. Definitive anatomical classification was determined according to the Standardization of Uveitis Nomenclature (SUN) Working Group, by reviewing the whole follow-up period [9].

The cause of uveitis was determined after the diagnostic examinations were completed. The diagnosis of definitive ocular sarcoidosis was given to patients that had histologically proven evidence and in all other cases; the criteria from the International Workshop on Ocular Sarcoidosis (IWOS) were used [10]. For the diagnosis of tuberculosis (TB)-associated uveitis, a positive culture for mycobacteria in any fluid/tissue sample was needed. Patients with a positive tuberculin skin test (Mantoux test) or interferon gamma release assay (IGRA) test with otherwise unexplained uveitis and no other indications of active tuberculosis were labeled as of unknown origin. All other specific diagnoses were performed according to current diagnostic criteria [10–16].

All statistical analyses were performed using SPSS software (version 22.0, Chicago, IL, USA) and a *P* value of < 0.05 was considered statistically significant. Specific groups were categorized as mentioned above and compared with each other according to gender, anatomical localization of uveitis, age, and etiology. Continuous variables were described by mean and range, categorical variables with proportions, and compared using the Mann-Whitney *U* test. Categorical variables were compared using the chi-square test or Fisher’s exact test.

## Results

The results of ESR and CRP measurements are shown in Table 1. Specific diagnoses in our cohort are depicted in the Supplemental Table. A majority of patients was diagnosed with associated non-infectious systemic diseases (59/174, 34%) and had non-anterior uveitis (141/174, 81%). Slight female preponderance was observed (96/174, 55%).

**Table 1:** Erythrocyte sedimentation rate and C-reactive protein of patients with a first episode of active uveitis of unknown cause

	Total *N* = 174	ESR^a^			CRP^a^		
		< 20 (*N* = 101)	20–59 (*N* = 57)	≥ 60 (*N* = 16)	< 10 (*N* = 131)	10–59 (*N* = 33)	≥ 60 (*N* = 10)
Total	174	101/174 (58%)	57/174 (33%)	16/174 (9%)	131/174 (75%)	33/174 (19%)	10/174 (6%)
Diabetes mellitus	17/174 (10%)	5/17 (29%)	9/17 (53%)	3/17 (18%)	12/17 (71%)	5/17 (29%)	0
Immune suppressive medication^b^	17/174 (10%)	5/17 (29%)	7/17 (41%)	5/17 (29%)	12/17 (71%)	2/17 (12%)	3/17 (18%)
Human immunodeficiency virus positivity^c^	11/174 (6%)	2/11 (18%)	5/11 (45%)	4/11 (36%)	10/11 (91%)	0	1/11 (9%)
Anatomical localization							
Anterior	33/174 (19%)	18/33 (55%)	10/33 (30%)	5/33 (15%)	23/33 (70%)	8/33 (24%)	2/33 (6%)
Intermediate	2/174 (1%)	1/2 (50%)	1 (50%)	0	2/2 (100%)	0	0
Posterior	45/174 (26%)	32/45 (71%)	12/45 (27%)	1/45 (2%)	37/45 (82%)	8/45(18%)	0
Panuveitis	86/174 (49%)	48/86 (56%)	30/86 (35%)	8/86 (9%)	65/86 (76%)	15/86(17%)	6/86 (7%)
Scleritis	8/174 (5%)	2/8 (25%)	4/8 (50%)	2/8 (25%)	4/8 (50%)	2/8 (25%)	2/8 (25%)
Non-infectious systemic disease	59/174 (34%)	33/59 (56%)	20/59 (34%)	6/59 (10%)	39/59 (66%)	14/59 (24%)	6/59 (10%)
Infectious uveitis	38/174 (22%)	20/38 (53%)	11/38 (29%)	7/38 (18%)	31/38 (82%)	4/38 (11%)	3/38 (8%)
Established clinical entity	24/174 (14%)	14/24 (58%)	8/24 (33%)	2/24 (8%)	17/24 (71%)	7/24 (29%)	0
Unknown	53/174 (30%)	34/53 (64%)	18/53 (34%)	1/53 (2%)	44/53 (83%)	8/53 (15%)	1/53 (2%)

*ESR* erythrocyte sedimentation rate, *CRP* C-reactive protein

^a^ESR had to be determined < 2 weeks of onset, CRP within < 1 week of onset

^b^Indicated for other causes than uveitis

^c^HIV was tested in 62 patients, out of which 11/62 (18%) were found positive

Immunosuppressive medication (required for other causes than uveitis) was used by 17/174, 10% patients. Patients suffering from diabetes mellitus (DM) and patients using immunosuppressive medication more often had elevated ESR values (*P* = 0.018 for both, chi-square test), compared to the remainder of patients. No significant differences in ESR and CRP levels were found for gender, race, localization, or laterality of uveitis (all *P* values > 0.05, chi-square test). Furthermore, elevated values of ESR and/or CRP were not significantly associated with any of the etiologic categories.

Concordance and discrepancies between ESR and CRP are depicted in Table 2. A majority of patients had both ESR and CRP values within the normal limits (91/174, 52%). Elevation of only one of the parameters was seen in 50/174, 29%. Elevated levels of both parameters were found in 33/174, 19% patients.

**Table 2:** Concordance and discrepancies in erythrocyte sedimentation rate and C-reactive protein in patients with uveitis

	Total *N* = 174	Concordant results ESR and CRP^a^		Discrepant results ESR and CRP^a^	
		CRP < 10 mg/L and ESR < 20 mm/h *N* = 91	ESR ≥ 20 mm/h and CRP ≥ 10 mg/L *N* = 33	ESR ≥ 20 mm/h but CRP < 10 mg/L *N* = 40	CRP ≥ 10 mg/L but ESR < 20 mm/h *N* = 10
Age at onset of uveitis (years) Mean (±SD)	45.8 (± 17.1)	43.1 (± 17.3)	5.9 (± 15.6)	51.7 (± 15.9)	46.6 (± 20.7)
Localization					
Anterior uveitis	33/174 (19%)	16/33 (48%)	8/33 (24%)	7/33 (21%)	2/33 (6%)
Intermediate uveitis	2/174 (1%)	1/2 (50%)	0	1/2 (50%)	0
Posterior uveitis	45/174 (26%)	29/45 (64%)	5/45 (11%)	8/45 (18%)	3/45 (7%)
Panuveitis	86/174 (49%)	44/86 (51%)	17/86 (20%)	21/86 (24%)	4/86 (5%)
Scleritis	8/174 (5%)	1/8 (13%)	3/8 (38%)	3/8 (38%)	1/8 (13%)
Laterality					
Unilateral	83/174 (48%)	43/83 (52%)	15/83(18%)	20/83 (24%)	5/83 (6%)
Bilateral	91/174 (52%)	48/91 (53%)	18/91 (20%)	20/91 (22%)	5/91 (5%)
Gender					
Females	96/174 (55%)	50/96 (52%)	16/96 (17%)	25/96 (26%)	5/96 (5%)
Males	78/174 (45%)	41/78 (53%)	17/78 (22%)	15/78 (19%)	5/78 (6%)
Race					
Caucasian	110/174 (63%)	59/110 (54%)	22/110 (20%)	23/110 (21%)	6/110 (5%)
Non-Caucasian	64/174 (37%)	32/64 (50%)	11/64 (17%)	17/64 (27%)	4/64 (6%)
Non-infectious systemic disease	59/174 (34%)	28/59 (47%)	15/59 (25%)	11/59 (19%)	5/59 (8%)
Sarcoidosis^b^	*24/59 (41%)*	10/24 (42%)	6/24 (25%)	7/24 (29%)	1/24 (4%)
HLA B27-associated uveitis	10/59 (17%)	6/10 (60%)	2/10 (20%)	0	2/10 (20%)
Miscellaneous^c^	25/ 59 (42%)	12/25 (48%)	7/25 (28%)	4/25 (16%)	2/25 (8%)
Infectious	38/174 (22%)	17/38 (45%)	4/38 (11%)	14/38 (37%)	3/38 (8%)
Toxoplasmosis	11/38 (29%)	9/11 (82%)	0	2/11 (18%)	0
;Endogenous endophthalmitis	7/38 (18%)	1/7 (14%)	2/7 (29%)	1/7(14%)	3/7 (43%)
;Miscellaneous^d^	20/38 (53%)	7/20 (35%)	2/20 (10%)	11/20 (55%)	0
Established clinical entity^e^	24/174 (14%)	13/24 (54%)	6/24 (25%)	4/24 (17%)	1/24 (4%)
Unknown	53/174 (30%)	33/53 (62%)	8/53 (15%)	11/53 (21%)	1/53 (2%)

*ESR* erythrocyte sedimentation rate, *CRP* C-reactive protein, *SD* standard deviation, *HLA B27* human leukocyte antigen B27

^a^ESR had to be determined < 2 weeks of onset, CRP within < 1 week of onset

^b^17/24 (71%) of sarcoidosis patients was biopsy confirmed

^c^Including patients with Vogt-Koyanagi-Harada syndrome (*N* = 6), multiple sclerosis (*N* = 4), Behçet’s disease (*N* = 3), inflammatory bowel disease (*N* = 3), granulomatosis with polyangiitis (*N* = 2), reactive arthritis with uveitis (*N* = 2), acute disseminated encephalomyelitis (*N* = 1), Kikuchi disease (N = 1), relapsing polychondritis (*N* = 1), systemic lupus erythematosus (*N* = 1), systemic vasculitis not otherwise specified (*N* = 1)

^d^Including varicella-zoster virus (*N* = 5), cytomegalovirus (N = 4), syphilis (*N* = 4), herpes simplex virus (*N* = 3), rubella virus (*N* = 2), bartonella (*N* = 1), tuberculosis (*N* = 1)

^e^Including patients with acute multifocal posterior placoid pigment epitheliopathy (*N* = 3), birdshot chorioretinopathy (*N* = 2), toxic uveitis (*N* = 2), post-traumatic uveitis (*N* = 2), sympathetic ophthalmia (*N* = 1), serpiginous choroidopathy (*N* = 1), purtscher-like retinopathy (*N* = 1), Fuchs heterochromic uveitis syndrome (*N* = 1), punctate inner choroidopathy (*N* = 1). The masquerade syndromes including lymphoma (*N* = 3), macular drusen (*N* = 2), human immunodeficiency virus–related microangiopathy (*N* = 1), macular dystrophy (*N* = 1), uveitis suspected to be caused by bacillus Calmette-Guérin intravesical immunotherapy for bladder cancer (*N* = 1), cotton wool spots (*N* = 1), Coats disease (*N* = 1)

The median ESR and CRP of patients with uveitis of established cause were higher than the median ESR and CRP of patients with unknown uveitis (17.0 mm/h, range 1–120 mm/h vs. 11.0 mm/h, range 1–140 mm/h for ESR and 3.4 mg/L, range 0.4–262.0 mg/L vs. 1.9 mg/L, range 0.3–229.0 mg/L for CRP; *P* = 0.015 for both, Mann-Whitney *U* test).

Out of 20 patients with either ESR ≥ 60 mm/h and/or CRP ≥ 60 mg/L, the cause of uveitis could be determined in 19/20. Fifteen had either non-infectious systemic disease or systemic infection, which was also a cause of uveitis (Table 3). The remaining five patients had uveitis limited to the eye, but had a concurrent systemic disorder, which explained their highly elevated ESR and/or CRP but was not related to the cause of uveitis (such as multiple myeloma in a patient with infectious uveitis).

**Table 3:** Patients exhibiting highly elevated erythrocyte sedimentation rate and/or C-reactive protein

	Erythrocyte sedimentation rate ≥ 60 mm/h and/or C-reactive protein ≥ 60 mg/L (*N* = 20)^a^
Associated systemic disease	2 Sarcoidosis 3 Reactive arthritis 1 Granulomatosis with polyangiitis, 1 Relapsing polychondritis 1 Behçet’s disease
Infectious disease	3 Endogenous endophthalmitis 2 Cytomegalovirus retinitis 1 Herpes simplex retinitis^b^ 1 Varicella-zoster anterior uveitis^b^ 1 Syphilis 1 Tuberculosis
Clinical entity	1 Masquerade syndrome^b^ 1 Post-traumatic uveitis^b^
Unknown	1^b^

^a^10 patients had ESR ≥ 60 mm/h, 4 patients had CRP ≥ 60 mg/L, and 6 patients both had ESR ≥ 60 mm/h and CRP ≥ 60 mg/L

^b^5 patients with disease limited to the eye had some other explanation for high elevation of ESR and/or CRP, namely multiple myeloma complicated with varicella-zoster uveitis (*N* = 1), sepsis in herpes simplex–associated uveitis (*N* = 1), inflammatory bowel disease with masquerade syndrome of drusen (*N* = 1), post-operative infection after orthopedic surgery in a patient with post-traumatic uveitis (*N* = 1), and a patient with fever, cachexia, and uveitis of unknown origin (*N* = 1)

A majority of patients with infectious uveitis had ESR and CRP values within the normal limits (17/38, 45%) or only ESR ≥ 20 mm/h (14/38, 37%), see Table 2. Discrepant results were more often noted in this group (17/38, 45% vs. 33/136, 24% *P* = 0.024, chi-square test). Patients with toxoplasmosis exhibited normal ESR values in 9/11, 82%, and all had CRP values within the normal limits. Patients with endogenous endophthalmitis exhibited CRP ≥ 10 mg/L in 5/7, 71%.

Patients with non-infectious uveitis commonly had ESR and CRP values that were both within the normal limits (28/59, 47%; Table 2). If elevated (*N* = 31), the parameters were most often elevated simultaneously (15/31, 48%). Of the patients with HLA B27-associated uveitis without systemic involvement, 2/8, 25% exhibited CRP ≥ 10 mg/L and 1/8, 13% had both ESR ≥ 20 mm/h and CRP ≥ 10 mg/L. Two patients had HLA B27-associated uveitis with systemic involvement of which one had high elevation of CRP ≥ 60 mg/L and ESR ≥ 20 mm/h and the other exhibited normal values. Sarcoidosis-associated uveitis showed a predominantly elevated ESR (13/24, 54%; in the range between 20 and 59 mm/h 11/13, 85%), while CRP was most often normal (17/24, 71%). Systemic involvement in sarcoidosis patients (hilar lymphadenopathy as seen on chest imaging) was present in 21/24 (88%); however, in only 4/21 (19%) of these, treatment was required.

The HIV was positive in 11/62, 18% tested patients. HIV-positive patients had more often elevated ESR values when compared to the remainder of patients (9/11, 82% vs. 64/163, 39%, *P* = 0.009, chi-square test). An infectious cause for uveitis was found in 9/11, 82% HIV-positive patients, out of which 4/9, 44% had CMV retinitis and 4/9, 44% had syphilitic uveitis. ESR ≥ 60 mm/h together with HIV positivity was observed in 4/11, 36% (2 with CMV retinitis, one with syphilitic uveitis, and one with sarcoidosis-associated uveitis). Only one of the HIV-positive patients exhibited normal values of both ESR and CRP; this patient was diagnosed with CMV retinitis.

## Discussion

In this retrospective, cross-sectional study, the majority of patients with a first active episode of uveitis of unknown origin presented with normal ESR and CRP values. Moreover, no significant relationship between the levels of these biomarkers and specific causes of uveitis was found.

Earlier investigations of ESR and CRP in uveitis patients also demonstrate normal values in a majority of patients with anterior uveitis, but none of these previous studies defined the time window in which ESR and CRP were determined in relation to the onset of uveitis, while both biomarkers are susceptible for changes within short periods [5, 8, 17]. Biomarkers like ESR and CRP are commonly assessed during the diagnostic workup of new uveitis patients for potential detection of infections or systemic immune-mediated disease-causing uveitis [1–4, 18–21]. Elevated levels of ESR are due to a higher plasma protein levels (e.g., fibrinogen, gamma globulins) and CRP is an acute phase protein released after tissue injury caused by infections or other sources of inflammation [8, 22].

Though a majority of patients with infectious uveitis in the present study exhibited normal ESR and CRP values, increased values were predominantly encountered in systemic infections. The high levels of ESR and CRP were found in patients with endogenous endophthalmitis, an ocular inflammation that occurs concurrently with bacteremia. In contrast, these inflammatory parameters were nearly always normal in patients with ocular toxoplasmosis, an intracellular parasite. Reactivation of these dormant parasites within the eye is not being accompanied by any systemic activity.

In HIV patients, the common hyperimmunoglobulinemia causes elevation of ESR (rather than a direct infectious trigger causing release of fibrinogen). In our series, highly elevated ESR was often seen in HIV-positive patients, and therefore, it might be therefore worthwhile to determine HIV status in patients with uveitis of unknown origin and unexplained high ESR [23]. Patients using immunosuppressive medications and those suffering from DM had more often elevated ESR values and the yield of these tests is therefore lower in these patients.

Elevated levels of ESR and CRP are common in systemic sarcoidosis patients, specifically in sarcoidosis-associated arthritis and erythema nodosum compared to other clinical presentations [24, 25]. Half of the patients with ocular sarcoidosis, however, had both normal ESR and CRP, which might reflect mild (or lack of) systemic involvement at the moment of onset of first uveitis attack [26]. This is also illustrated in the current series, where the majority of patients had very mild extraocular involvement which might also explain their predominantly normal ESR and CRP values.

In this study, follow-up measurements of ESR and/or CRP were not available, and consequently, we do not have information about the highest levels of ESR and CRP reached in individual patients. Including the highest levels of ESR and CRP could possibly expose some associations, which were not found in the present study. In addition, the changes of ESR and/or CRP might change in individual patients and might be associated with impeding uveitis activity. However, our main goal was to determine the diagnostic value of these parameters during the first stage of uveitis after presentation. Data on body mass index, which can influence the ESR and CRP values, were not available in our patients [27].

In conclusion, our study reflects that presence of uveitis alone is not sufficient to cause elevation of ESR and/or CRP as a majority of patients with a first uveitis episode had ESR and CRP values within the normal limits. In patients with highly elevated ESR and/or CRP, the presence of a systemic disease is very likely, and in consequence, the cause of uveitis is being established in a vast majority of cases.

## Electronic supplementary material

Supplementary Table (DOCX 14 kb)
