# Supplementary material for: Relevance of erythrocyte sedimentation rate and C-reactive protein in patients with active uveitis
Source: Graefes Arch Clin Exp Ophthalmol. 2018 Nov 8;257(1):175–80. doi: 10.1007/s00417-018-4174-7 (PMC6323081; doi:10.1007/s00417-018-4174-7)
Supplement: Supplementary file 1 — (DOCX 14 kb) [file 417_2018_4174_MOESM1_ESM.docx]

**Supplemental Table.** Demographics and baseline characteristics of patients with active uveitis of unknown cause at onset**.**

|  | **ESR/CRP performed^a^** |
| --- | --- |
| **Total no. of included patients** | 174 |
| **Age at onset of uveitis (years)**  *Mean (±SD)* | 45.8 *(±17.1)* |
| **Unilateral involvement** | 83/174 (48%) |
| **Bilateral involvement** | 91/174 (52%) |
| **Females** | 96/174 (55%) |
| **Males** | 78/174 (45%) |
| **Race** |  |
| *Caucasian* | 110/174 (63%) |
| *Non-Caucasian* | 64/174 (37%) |
| **Anatomical localization** |  |
| *Anterior* | 33/174 (19%) |
| *Intermediate* | 2/174 (1%) |
| *Posterior* | 45/174 (26%) |
| *Panuveitis* | 86/174 (49%) |
| *Scleritis* | 8/174 (5%) |
| **Non-infectious systemic disease** | **59/174 (34%)** |
| *Sarcoidosis*  *Biopsy-proven sarcoidosis*  *Presumed sarcoidosis* | *24/59 (41%)*  *17/24 (71%)*  *7/24 (29%)* |
| *HLA B27-associated uveitis* | 10/59 (17%) |
| *VKH- syndrome* | 6/59 (10%) |
| *MS* | 4/59 (7%) |
| *Behçet's disease* | 3/59 (5%) |
| *Miscellaneous*^b^ | 12/59 (20%) |
| **Infectious uveitis** | **38/174 (22%)** |
| *Toxoplasmosis* | 11/38 (29%) |
| *Endogenous endophthalmitis* | 7/38 (18%) |
| *VZV* | 5/38 (13%) |
| *Syphilis* | 4/38 (11%) |
| *CMV* | 4/38 (11%) |
| *HSV* | 3/38 (8%) |
| *Rubella* | 2/38 (5%) |
| *Bartonella* | 1/38 (3%) |
| *Tuberculosis (active)* | 1/38 (3%) |
| **Established clinical entity** | **24/174 (14%)** |
| *Masquerade^c^* | 10/24 (42%) |
| *AMPPE* | 3/24 (13%) |
| *BSCR* | 2/24 (8%) |
| *Miscellaneous*^d^ | 9/24 (38%) |
| **Unknown**  *QFT positive*  *QFT negative*  *QFT not performed* | **53/174 (30%)**  *7/53 (13%)*  *26/53 (49%)*  *20/53 (38%)* |

*ESR = erythrocyte sedimentation rate, CRP = C-reactive protein, SD = standard deviation, HLA B27 = human leukocyte antigen B27, VKH = Vogt-Koyanagi-Harada, MS = multiple sclerosis, VZV = varicella zoster Virus, CMV = Cytomegalovirus, HSV = herpes simplex virus, AMPPE = acute multifocal posterior placoid pigment epitheliopathy, BSCR = birdshot chorioretinopathy, QFT= quantiferon test.*

*^a^ ESR had to be determined <2 weeks of onset, CRP within <1 week of onset.*

*^b^ Including patients with inflammatory bowel disease (N=3), granulomatosis with polyangitis (N=2), reactive arthritis with uveitis (N=2), acute disseminated encephalomyelitis (N=1), kikuchi disease (N=1), relapsing polychondritis (N=1), systemic lupus erythematosus (N=1), systemic vasculitis not otherwise specified (N=1).*

*^c^ Including lymphoma (N=3), macular drusen (N=2), human immunodeficiency virus related microangiopathy (N=1), macular dystrophy (N=1), uveitis suspected to be caused by bacillus Calmette-Guérin intravesical immunotherapy for bladder cancer (N=1), cotton wool spots (N=1), Coats’ disease (N=1).*

*^d^ Including patients with toxic uveitis (N=2), post-traumatic uveitis (N=2), sympathetic ophthalmia (N=1), serpiginous choroidopathy (N=1), purtscher like retinopathy (N=1), Fuchs heterochromic uveitis syndrome (N=1), punctate inner choroidopathy (N=1).*
